# Synthesis of (*R*)-mandelic acid and (*R*)-mandelic acid amide by recombinant *E. coli* strains expressing a (*R*)-specific oxynitrilase and an arylacetonitrilase

**DOI:** 10.1007/s10529-020-02998-8

**Published:** 2020-09-16

**Authors:** Erik Müller, Olga Sosedov, Janosch Alexander David Gröning, Andreas Stolz

**Affiliations:** 1 Institut für Mikrobiologie, Universität Stuttgart, Allmandring 31, 70569 Stuttgart, Germany; 2 Present Address: Biochem Labor für chemische Analytik GmbH, Daimlerstr. 5B, 76185 Karlsruhe, Germany

**Keywords:** Biotransformations, Chiral synthesis, Nitrilase, Oxynitrilase, Hydroxynitrile lyase, Enzyme cascades

## Abstract

**Abstract:**

**Objectives:**

Chiral 2-hydroxycarboxylic acids and 2-hydroxycarboxamides are valuable synthons for the chemical industry.

**Results:**

The biocatalytic syntheses of (*R*)-mandelic acid and (*R*)-mandelic acid amide by recombinant *Escherichia coli* clones were studied. Strains were constructed which simultaneously expressed a (*R*)-specific oxynitrilase (hydroxynitrile lyase) from the plant *Arabidopsis thaliana* together with the arylacetonitrilase from the bacterium *Pseudomonas fluorescens* EBC191. In addition, recombinant strains were constructed which expressed a previously described acid tolerant variant of the oxynitrilase and an amide forming variant of the nitrilase. The whole cell catalysts which simultaneously expressed the (*R*)-specific oxynitrilase and the wild-type nitrilase transformed in slightly acidic buffer systems benzaldehyde plus cyanide preferentially to (*R*)-mandelic acid with ee-values > 95%. The combination of the (*R*)-specific oxynitrilase with the amide forming nitrilase variant gave whole cell catalysts which converted at pH-values ≤ pH 5 benzaldehyde plus cyanide with a high degree of enantioselectivity (ee > 90%) to (*R*)-mandelic acid amide. The acid and the amide forming catalysts also converted chlorinated benzaldehydes with cyanide to chlorinated mandelic acid or chlorinated mandelic acid amides.

**Conclusions:**

Efficient systems for the biocatalytic production of (*R*)-2-hydroxycarboxylic acids and (*R*)-2-hydroxycarboxamides were generated.

## Introduction

Organic nitriles are natural products and are also synthesized in huge amounts by the chemical industry. There are several enzymes known which either form or convert organic nitriles and there is considerable interest in applying these enzymes in biotechnology. Currently, the major interest in nitrile forming enzyme lies in oxynitrilases (hydroxynitrile lyases) which can form chiral 2-hydroxynitriles from aldehydes (or ketones) and cyanide (Bracco et al. 2016). In addition, there are two groups of nitriles converting enzymes intensively studied which possess a considerable potential for biotransformation reactions. These are nitrilases which are able to hydrolyse nitriles to the corresponding carboxylic acids (and ammonia) and nitrile hydratases which convert nitriles to the corresponding amides (Martinková and Křen 2010).

**Fig. 1:**
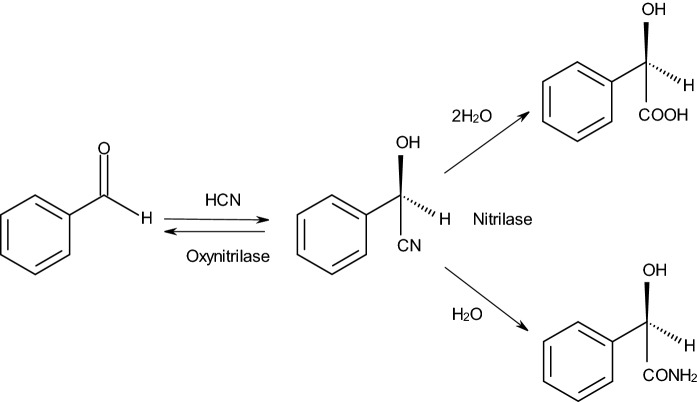
Enantioselective synthesis of (*R*)-mandelic acid and (*R*)-mandelic acid amide by a combination of an enantioselective oxynitrilase and a non-selective nitrilase or an enzyme with nitrile hydratase activity

In the last years, the combination of (*S*)-specific oxynitrilases with nitrilases or nitrile hydratases has been described for the synthesis of chiral (*S*)-2-hydroxycarboxylic acids or (*S*)-2-hydroxycarboxamides. In these systems, the (*S*)-specific oxynitrilase from the cassava plant (*Manihot esculenta*) was combined with nitrilases or nitrile hydratases either in-vitro (often in the form of cross-linked enzyme aggregates-“CLEAs”) or in-vivo by using recombinant organisms (*Escherichia coli* or *Pichia pastoris*) which simultaneously expressed oxynitrilase and nitrilase activities (Fig. 1). These systems allowed the efficient synthesis of chiral 2-hydroxycarboxylic acids and 2-hydroxycarboxamides from non-chiral aldehydes (or ketones) and cyanide (van Rantwijk and Stolz 2015).

In the present study, it was analysed if it was possible to create an isofunctional system for the synthesis of (*R*)-2-hydroxycarboxamides and (*R*)-2-hydroxycarboxylic acids by the combination of an (*R*)-specific oxynitrilase with enzymes demonstrating nitrilase or nitrile hydratase activities.

## Materials and methods

### Bacterial strains, plasmids, and culture conditions

*Escherichia coli* JM109 was used as host strain for all plasmids. The strain was routinely cultivated at 30 °C on Luria–Bertani Broth (LB) (with antibiotics if required).

Plasmid pIK9 codes for a His-tagged variant of the nitrilase from *P. fluorescens* EBC191 (Kiziak et al. 2005).

### DNA handling

All DNA-manipulation techniques were basically performed as described by Sambrook et al. (1989). Plasmids were isolated using a “NucleoSpin Plasmid Kit” (Macherey & Nagel).

### Cloning of the gene coding for the hydroxynitrile lyase from *Arabidopsis thaliana* under the control of the *rhaP*_BAD_ promotor

The DNA-sequence coding for the hydroxynitrile lyase from *A. thaliana* (*At*HNL) (NCBI No AY093714) was ordered from the “Arabidopsis Biological Resource Center” (Ohio State University, Columbus, OH, USA) (Kayoko et al. 2003). The gene coding for *At*HNL (supplied in the vector plasmid pUN151) was amplified by PCR by using the primer pair AtHNL-Nde-5´ (GAAATTCCATATGGAGAGGAAACATCACTTCG) and AtHNL-Hind-3´(ACGCAAGCTTACATATAATCGGTGGCAATAGCAGAG) which introduced restriction sites for NdeI and HindIII, respectively (underlined). The amplified DNA-fragment was cut with NdeI and HindIII.

Plasmid pJOE5361.1, which is a derivative of plasmid pAW229 carrying an (*S*)-oxynitrilase gene (Sosedov et al. 2009; Wilms et al. 2001) was cut with NdeI and HindIII. The (*S*)-oxynitrilase gene was removed by agarose gel electrophoresis and the vector DNA ligated with the DNA-fragment coding for the *At*HNL (also cut with NdeI and HindIII). Thus, plasmid pSOM4 was generated which expressed *At*HNL under the control of the *rhaP*_BAD_ promotor.

### Generation of an expression plasmid synthesizing an acid stabilized variant of the hydroxynitrile lyase from *Arabidopsis thaliana*

A synthetic variant of the gene coding for *At*HNL which encoded for all amino acid exchanges (P48Q, Q50E, A51Q, E53N, K60E, E64T, K67E, I93R, E138T, R140I, N141T) described by Okrob et al. (2012) was synthesized and delivered in a recombinant plasmid by a commercial supplier (Invitrogen). This plasmid was cleaved with NdeI and HindIII and plasmid pEM4 generated by replacing the gene coding for the wild-type AtHNL in pSOM4 by the synthetic gene.

### Preparation of whole cells catalysts

Precultures of the recombinant *E. coli* strains [in LB-medium with the relevant antibiotic(s)] were transferred (1:100 v/v) to LB-media (+ antibiotics) containing 0.2% (w/v) l-rhamnose to induce the genes encoding the hydroxynitrile lyase and the nitrilase. The cultures were grown at 100 rpm for 18 h at 30 °C and the cells harvested by centrifugation in the late exponential or early stationary growth phase. The cells were either used immediately or stored frozen at – 70 °C before usage.

### Biotransformation assays

The aliquots were thawed, the cells washed in Na-citrate buffer (100 mM, pH 5) and finally resuspended to optical densities (OD_600nm_) of 0.2–20 in Na-citrate buffer (100 mM, pH 5). Resting cells (970 µL) were incubated at 30 °C in and benzaldehyde (1 µL) added. The reaction mixtures were shaken (750 rpm) for about 3 min and finally, a KCN-solution added (30 µL from a freshly prepared 0.68 M stock solution). At different time intervals, aliquots (90 µL each) were removed, the reactions stopped by centrifugation (21,000 rpm, 2 min) and the concentrations of substrates and products in the supernatants quantified by HPLC.

One unit of enzyme activity was defined as conversion (or formation) of 1 µmol of substrate (or product) per min.

### Preparation of cell extracts

Cell extracts were prepared by sonification of cooled cell suspensions (Sonoplus HD200, MS 73 Sontrode, 4 pulses á 30 s). Cells and cell debris were removed in a cooled Eppendorf centrifuge (14,000 rpm, 4 °C, 30 min).

### Analytical methods

The concentrations of benzaldehyde, mandelonitrile, mandelic acid amide, mandelic acid and their chlorinated derivatives were determined by HPLC. For the achiral analysis of the substrates and products, a Lichrospher RP18 column was used (250 $\times$ 4 mm, 5 µm particles). The compounds were eluted using a mobile phase which initially (t = 0–25 min) consisted of 40% (v/v) methanol, 59.7% (v/v) water, and 0.3% (v/v) H_3_PO_4_. Subsequently, a linear increase in the methanol concentration to 60% (v/v) methanol was applied (t = 25–35 min). The average flow rate was 0.3 mL/min.

The enantiomeric composition of the chiral compounds was analysed by using a Chiral-HSA column (150 $\times$ 4 mm; ChromTech AB, Hägersten, Sweden). The mobile phase consisted of 95.5% (v/v) sodium phosphate buffer (50 mM, pH 7.0) plus 4.5% (v/v) acetonitrile. The flow rate was set to 0.5 mL/min.

The separated compounds were detected spectrophotometrically at 210 nm.

### Chemicals

(*R*)- and (*S*)-mandelic acid amide were obtained from Activate Scientific (Prien, Germany).

## Results

### Identification of a suitable (*R*)-oxynitrilase

(*R*)-specific oxynitrilases have initially been described in plants belonging to the family *Rosaceae* and most biotransformation reactions described in the literature have been performed with the (*R*)-oxynitrilase from bitter almonds (*Prunus amygdalus*) (Bracco et al. 2016; Griengl et al. 2000). (*R*)-specific oxynitrilases have also been detected in plants belonging to different families, e.g. in flax (*Linum usitatissimum*), passion fruit (*Passiflora edulis*), mouse-ear cress (*Arabidopsis thaliana*), and the ferns *Phlebodium aureum* and *Davallia tyermannii* (Albrecht et al. 1993; Andexer et al. 2007; Lanfranchi et al. 2017; Motojima et al. 2017; Wajant et al. 1995). More recently, (*R*)-specific oxynitrilases have also been described from bacteria, such as *Pseudomonas mephitica*, *Burkholderia phytofirmans*, *Granulicella tundricola*, and *Acidobacterium capsulatum* (Hajnal et al. 2013; Hussain et al. 2012; Wiedner et al. 2014).

The (*R*)-specific oxynitrilases from the members of the *Rosaceae* are flavin-containing glycoproteins and are therefore problematic to express in recombinant systems. In contrast, the oxynitrilases from *A. thaliana*, *L. usitatissimum* and *P. aureum* contain neither carbohydrate- nor flavin-residues (Andexer et al. 2007; Trummler and Wajant 1997; Wajant et al. 1995). The enzyme from *A. thaliana* was chosen for the construction of the intended cascade reaction, as it has been intensively studied and it has been demonstrated that it converts a broad range of aldehydes in the presence of cyanide enantioselectively to the corresponding (*R*)-2-hydroxynitriles (Andexer et al. 2007, 2012; Okrob et al. 2011). Furthermore, a variant of the enzyme has been described which showed *in vitro* enhanced acid stability (Okrob et al. 2012).

### Construction of whole cell catalysts which express the wild-type and the acid-tolerant form of the (*R*)-specific oxynitrilase from *A. thaliana*

The gene coding for the oxynitrilase from *A. thaliana* was cloned in plasmid pJOE5361.1 under the control of the rhamnose-inducible *rhaP*_BAD_ promotor and *E. coli* JM109 transformed with this construct (pSOM4; see "Materials and methods" section). The intended biotransformation reactions required rather acidic conditions to prevent the racemization of the intermediately formed (*R*)-mandelonitrile. Therefore, it was tested if the application of a variant of *At*HNL (*At*HNLv2) which has been described by Okrob et al. (2012) as more acid tolerant than the wild-type enzyme resulted in increased activities and/or enantioselectivities. For that reason, the gene encoding for the wild-type AtHNL in plasmid pSOM4 was replaced by a synthetic gene coding for *At*HNLv2 and the resulting plasmid designated as pEM4 (see "Materials and methods" section).

### Functional expression of the (*R*)-specific oxynitrilase from *A. thaliana* and its acid tolerant variant in *E. coli*

*Escherichia coli* JM109(pSOM4) and *E. coli* JM109(pEM4) were grown in LB-medium with chloroamphenicol (20 µg/mL) and the synthesis of *At*HNL or *At*HNLv2 induced by the addition of rhamnose (0.2% w/v) as described in the "Materials and methods" section. The oxynitrilase activity was determined in cell extracts spectrophotometrically by determining the formation of benzaldehyde from mandelonitrile (basically as described by Ueatrongchit et al. 2009). Thus, in the cell extracts from *E. coli* JM109(pSOM4) and *E. coli* JM109(pEM4) oxynitrilase activities of 2.0 and 2.9 U/mg of protein were found.

The oxynitrilase activity was also confirmed with whole cells in the synthetic direction. Cells of *E. coli* JM109(pSOM4) were harvested by centrifugation at the end of the exponential growth phase and resuspended at 30 °C in Na-citrate buffer (100 mM, pH 4.5). Then, 10 mM benzaldehyde (from a 1 M methanolic stock solution) and 20 mM KCN (from a 2 M stock solution in H_2_O) were added, the reactions analysed by HPLC, and compared to a control experiment under the same conditions but without resting cells. In the presence of the resting cells, mandelonitrile was formed almost nine-times more rapidly than in the control experiment. This demonstrated that the resting cells indeed exhibited an oxynitrilase activity.

### Construction of “bienzymatic catalysts” which simultaneously express the (*R*)-specific oxynitrilase from *A. thaliana* or its acid tolerant variant together with the nitrilase from *Pseudomonas fluorescens* EBC191

To obtain “bienzymatic catalysts” with the ability to convert benzaldehyde plus cyanide to (*R*)-mandelic acid, *E. coli* JM109(pSOM4) and *E. coli* JM109(pEM4) were transformed with plasmid pIK9, which codes for the (almost non-enantioselective) nitrilase from *P. fluorescens* EBC191 (Kiziak et al. 2005). This resulted in *E. coli* JM109(pSOM4)(pIK9) and *E. coli* JM109(pEM4)(pIK9) which carried the genes coding for the (*R*)-oxynitrilase (or its acid tolerant variant) and the nitrilase on two compatible plasmids under the control of the same *rhaP*_BAD_ promotor. Thus, it was possible to induce both recombinant enzymes simultaneously by the addition of rhamnose.

**Fig. 2:**
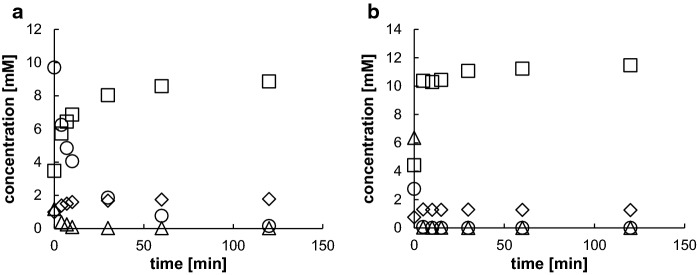
Conversion of benzaldehyde and KCN by resting cells of **a** *E. coli*(pSOM4)(pIK9) and **b** *E. coli*(pEM4)(pIK9). The preparation of the resting cells (OD_600nm_ = 10) and the reaction conditions were described in the "Materials and methods" section. The concentrations of benzaldehyde (open circle), mandelonitrile (open triangle), mandelic acid amide (rhombus), and mandelic acid (open square) were quantified by HPLC

The intended biotransformation reactions required rather acidic conditions, as the intermediately formed chiral 2-hydroxynitriles rapidly isomerize under neutral conditions (Sosedov et al. 2009). Hence, the relevant enzyme activities were induced in the *E. coli* strains by the addition of rhamnose and resting cells suspended in Na-citrate buffer at pH 5 (see "Materials and methods" section). The biotransformations were started by the addition of benzaldehyde and KCN and the reactions analyzed by HPLC. Both whole cell catalysts converted benzaldehyde and cyanide mainly to mandelic acid and small amounts of mandelic acid amide. The cells of *E. coli* JM109(pEM4)(pIK9) showed an almost three times higher rate for the formation of mandelic acid than *E. coli* JM109(pSOM4)(pIK9) (Fig. 2). The analysis of the mandelic acid formed by chiral HPLC demonstrated that in both systems almost exclusively (R)-mandelic acid was formed (ee > 95%). This clearly demonstrated that the (*R*)-oxynitrilase and the nitrilase were active in the whole cell catalysts because it was previously shown that the nitrilase from *P. fluorescens* EBC191 converts mandelonitrile in the absence of an oxynitrilase activity only with much lower enantioselectivity to (*R*)-mandelic acid (with an ee of about 30%) and additionally forms higher amounts of mandelic acid amide from racemic mandelonitrile (Kiziak et al. 2007; Stolz et al. 2019).

In the following, it was tested if a difference between *E. coli* JM109(pSOM4)(pIK9) and *E. coli* JM109(pEM4)(pIK9) for the conversion of benzaldehyde plus cyanide at more acidic pH-values could be observed. Therefore, the biotransformation reactions were repeated in Na-citrate buffers at pH 4.0, 4.5, 5.0, and 5.5, but the whole cell catalyst expressing AtHNLv2 did not show any advantage compared to the cells which synthesized the wild-type oxynitrilase.

### Construction of “bienzymatic catalysts” with the ability to synthesize (*R*)-mandelic acid amide

**Fig. 3:**
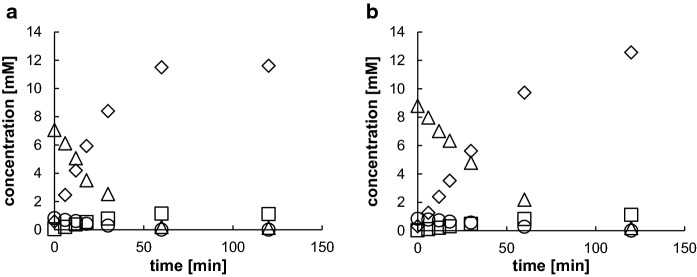
Conversion of benzaldehyde and KCN by **a** *E. coli* JM109(pSOM4)(pIK9/W188K) and **b*** E. coli* JM109(pEM4)(pIK9/W188K). The bacterial cultures were grown and the biotransformation experiments were performed as described in the "Materials and methods" section using whole cell suspensions with an OD_600nm_ = 20 in Na-citrate buffer (100 mM, pH 5). The concentrations of benzaldehyde (open circle), mandelonitrile (open triangle), mandelic acid (open square), and mandelamide (rhombus) were determined by HPLC

Previously, several variants of the nitrilase from *P. fluorescens* EBC191 have been obtained which form increased amounts of mandelic acid amide from mandelonitrile (Kiziak et al. 2007, Kiziak and Stolz 2009; Sosedov et al. 2010; Sosedov and Stolz 2014). In the course of these investigations, the nitrilase variant Trp188Lys was generated which converted racemic mandelonitrile to more than 90% of mandelic acid amide (Sosedov and Stolz 2015). Therefore, *E.coli* JM109(pSOM4) and *E. coli* JM109(pEM4) were transformed with plasmid pIK9/W188K which encodes the relevant nitrilase variant. Subsequently, these “bienzymatic catalysts” were incubated with benzaldehyde and KCN and the reactions analysed by HPLC. These whole cell catalysts formed almost exclusively mandelic acid amide and only traces of mandelic acid (Fig. 3). In contrast to the catalysts synthesizing the wild-type nitrilase (see Fig. 2), in these experiments significant amounts of mandelonitrile were intermediately formed. This indicated that in the constructs expressing the Trp188Lys-variant, the oxynitrilase activities were much higher than the amide forming activities. Nevertheless, after prolonged incubation times the benzaldehyde was almost stoichiometrically converted to mandelic acid amide although only with a low degree of enantioselectivity (ee-values for (*R*)-mandelic acid amide of 40–50%).

### Enantioselective formation of (*R*)-mandelic acid amide from benzaldehyde and cyanide

**Fig. 4:**
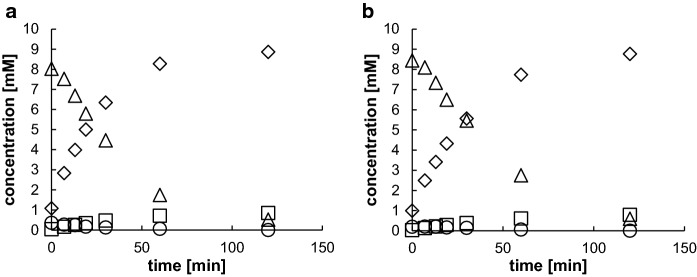
Conversion of benzaldehyde and KCN by **a** *E. coli* JM109 (pSOM4)(pIK9|W188K) and **b** *E. coli* JM109 (pEM4)(pIK9|W188K). The freshly harvested cells were resuspended in 100 mM Na-citrate buffer (pH 4) to an OD_600nm_ of 20. The turn-over experiments were performed as described in the "Materials and methods" section. The concentrations of (open circle) benzaldehyde, (open triangle) mandelonitrile, (open square) mandelic acid (open square) mandelic acid amide (open rhombus) were determined by HPLC

The results described above suggested that at the used pH of 5.5 parts of the intermediately accumulating mandelonitrile racemized and that this resulted in the low enantioselectivity of the reactions. Therefore, to suppress the chemical racemization, the reaction was repeated at lower pH-values. *E.coli* JM109(pSOM4)(pIK9/W188K) and *E.coli* JM109(pEM4)(pIK9/W188K) were cultivated as described above and freshly harvested cells suspended to an increased optical density (OD_600nm_ = 20) in Na-citrate buffer at pH 4.0. Both “bienzymatic catalysts” converted benzaldehyde almost stoichiometrically to mandelic acid amide (Fig. 4). The analysis of the samples by chiral HPLC showed that only (*R*)-mandelic acid amide (and no (*S*)-mandelic acid amide) could be detected.

### Conversion of chlorinated benzaldehydes by the “bienzymatic catalysts”

Chlorinated derivatives of mandelic acid and mandelic acid amide are interesting intermediates for the pharmaceutical industry (He at al. 2010; Wang et al. 2014; Zhang et al. 2012). Therefore, the conversion of 2-chloro-, 3-chloro-, and 4-chlorobenzaldeyde by the “bienzymatic catalysts” was studied.

*Escherichia coli* JM109(pEM4)(pIK9) was incubated in Na-citrate buffer (100 mM, pH 5) with benzaldehyde, 2-chloro-, 3-chloro-, or 4-chlorobenzaldehyde (10 mM each) plus KCN (20 mM) and the formation of mandelic acid and the respective chlorinated mandelic acids analysed by HPLC. The resting cells converted all three chlorinated benzaldehydes to the corresponding chlorinated mandelic acids. The relative rates for the formation of mandelic acid, 2-chloro-, 3-chloro-, and 4-chloromandelic acids were 100:5:39:21.

In the case of the chlorinated mandelic acids, the racemic compounds and the respective (*R*)-enantiomers were commercially available. The chiral analysis of the biotransformation experiments indicated that during the conversion of 3-chloro- and 4-chlorobenzaldehyde only the (*R*)-enantiomers of 3-chloro- and 4-chloromandelic acids were formed.

The analyses of the reactions performed by *E. coli* JM109(pEM4)(pIK9/W188K) were much more difficult to interpret as the corresponding chlorinated mandelic acid amides were commercially not available. Nevertheless, the biotransformation could be explained under the assumption that the chlorinated compounds eluted from the column in the same order as the non-chlorinated compounds (= amide before acid before nitrile before aldehyde). Thus, it became clear that 3-chlorobenzaldehyde was converted to the 3-chloromandelic acid amide with a similar rate as benzaldehyde to the mandelic acid amide. In contrast, 4-chlorobenzaldehyde was converted only with about 20% of the rate found for 3-chlorobenzaldehyde and there was almost no conversion of 2-chlorobenzaldehyde.

## Discussion

The “bienzymatic catalysts” described in the present manuscript in combination with the previously constructed recombinant strains which simultaneously express the (*S*)-specific oxynitrilase from cassava together with the nitrilase(variants) allow the facile synthesis of a wide range of chiral hydroxycarboxylic acids and amides from simple precursors. This can be deduced from the “proof of principle” experiments described in this and previous publications for the “bienzymatic catalysts” and the substrate ranges of the used nitrilase and oxynitrilases which demonstrated for all three enzymes (the oxynitrilases from cassava and *Arabidoposis thaliana* and the nitrilase from *P. fluorescens* EBC 191) rather broad substrate specifities (Andexer et al. 2007; Baum et al. 2012; Brunner et al. 2018; Bühler et al. 2003; Kiziak et al. 2005, Sosedov et al. 2009).

It might appear at the first sight unnecessary to construct (*R*)-specific “bienzymatic catalysts” for the synthesis of (*R*)-hydroxycarboxylic acids from aldehydes and cyanide as the synthesis of (*R*)-mandelic acid from mandelonitrile by enantioselective nitrilases has been described several times. Unfortunately, this procedure is limited as there are only a few highly enantioselective nitrilases and the enantioselectivity of these enzymes seems usually be limited to the conversion of mandelonitrile and few derivatives (Banerjee et al. 2006; Kaul et al. 2004; Martinková and Křen 2018; Wang et al. 2014; Yamamoto et al. 1992; Zhang et al. 2012).

There is a considerable interest in “green chemistry” to find new synthetic (enantioselective) amide forming reactions as these are of great importance for the chemical and pharmaceutical industry (Constable et al. 2007). Furthermore, (substituted) mandelic acid amide(s) are building blocks of several antibiotics, fungicides, and antioxidatives (Cederbaum et al. 2004; Cole 1969; Ley and Bertram 2001). Therefore, it was disappointing that all previously studied variants of the nitrilase from *P. fluorescens* EBC191 which formed significant amounts of mandelic acid amide only formed (*S*)-mandelic acid amide with a rather low-degree of enantioselectivity (Stolz et al. 2019). In contrast, the efficient synthesis of (*R*)-mandelic acid amide shown in the present study by the system expressing the (*R*)-specific oxynitrilase and the amide forming nitrilase variant, opens new perspectives for the enantioselective synthesis of (*R*)-hydroxycarboxamides. Surprisingly, there seems to be only one previous publication which describes a biocatalytic access to (*R*)-mandelic acid amide (Martinková and Křen 2018). This work described the asymmetric amidation of (*R*,*S*)-mandelic acid by using a lipase (Yildirim and Tükel 2014).

It is known for a long time that the successful synthetic application of oxynitrilases for the synthesis of chiral α-hydroxynitriles (cyanohydrines) requires rather acidic conditions (or a large surplus of organic solvents) to prevent the chemical racemization of the formed chiral cyanohydrines (Bracco et al. 2016; Griengl et al. 2000). Unfortunately, the oxynitrilase from *A. thaliana* showed in vitro only insufficient acid stability and was therefore in aqueous media only of limited synthetic value. To circumvent this problem, in previous work, the reactions were performed with purified enzymes either in media with a rather low water content or by using an acid tolerant oxynitrilase variant (Okrob et al. 2011, 2012). In contrast to these studies, we used whole cell catalysts which recombinantly expressed the oxynitrilase activities. Surprisingly, during all our experiments in no case at any tested acidic pH-value a positive effect of the “acid-tolerant variant” on the conversion rates or catalyst stability was observed. This strongly indicated that in the cytoplasm of the whole cell system even the wild-type oxynitrilase is sufficiently protected from the acidic conditions in the medium. This observation might further extend the applicability of enantioselective oxynitrilases for synthetic applications.
